# Exclusive breastfeeding practices in relation to social and health determinants: a comparison of the 2006 and 2011 Nepal Demographic and Health Surveys

**DOI:** 10.1186/1471-2458-13-958

**Published:** 2013-10-14

**Authors:** Vishnu Khanal, Kay Sauer, Yun Zhao

**Affiliations:** 1 School of Public Health, Curtin University, Bentley, Australia; 2 Centre for Behavioural Research in Cancer Control, Curtin University, Perth, Western Australia, Australia; 3 Sauraha Pharstikar Village Development Committee, Ward 1, Rupandehi, Nepal

## Abstract

**Background:**

Exclusive breastfeeding (EBF) for the first six months can have a significant impact on reducing child morbidity and mortality rates. The objective of this study was to compare the determinants of and trends in EBF in infants ≤5 months from the 2006 and 2011 Nepal Demographic and Health Surveys.

**Methods:**

Data on mother/infant pairs having infants of ≤5 months from 2006 (n = 482) and 2011 (n = 227) were analysed. The EBF rate, determinants of EBF, and changes in EBF rates between the 2006 and 2011 surveys were examined using Chi-square test and multiple logistic regression.

**Results:**

The EBF rate for ≤5 months in 2006 was 53.2% (95% CI, 47.1%-59.3%) and 66.3% (95% CI, 56.6%-74.8%) in 2011. In 2006, infants ≤4 months were more likely to be EBF [(aOR) 3.086, 95% CI (1.825-5.206)] after controlling for other factors. A geographic effect was also found in this study, with the odds of EBF higher for infants from the Hills [aOR 3.426, 95% CI (1.568-7.474)] compared to those form the mountains. The odds of EBF were also higher for higher order infants [aOR 1.968, 95% CI (1.020-3.799)]. Infants whose fathers belonged to non-agricultural occupation were less likely to be provided with EBF. Infants who were delivered in the home were more likely to experience EBF [aOR 1.886; 95% CI (1.044-3.407)]. In 2011, infants of age ≤4 months were more likely [aOR 4.963, 95% CI (2.317-10.629)] to have been breastfed exclusively. While there was an increase in the EBF rate between 2006 and 2011 surveys, the significant increase was noticed only among the infants of four months [32.0%; 95% CI (19.9%-47.0%)] in 2006 to [65.5%; 95% CI (48.1-79.6)] in 2011.

**Conclusions:**

The proportion of infants who were EBF was higher in Nepal in 2011survey compared to 2006 survey; however, this is still below the recommended WHO target of 90%. Infant’s age, ecological region, parity and father’s occupation were associated with EBF. Further interventions such as peer counselling, antenatal counselling and involving fathers in the community to promote EBF in Nepal are recommended.

## Background

The World Health Organization (WHO) recommends exclusive breastfeeding (EBF) up to six months of age [1,2]. During this six-month period, no liquid, semisolid or solid food or breastfeeding substitute should be given to the infants except for medicine and/or oral rehydration solution. EBF has been considered beneficial to the health and wellbeing of infants and mothers [3]. A child who is EBF for six months is more likely to be protected from gastrointestinal infections, respiratory illness, morbidity and death [4-6]. EBF can also protect the child from atopic eczema [5,7], the risk of allergy, asthma, type II diabetes [8], leukaemia [9] and obesity in later life [3].

Rates of infant mortality (46 per 1000 live births) and an under five mortality (54 per 1000 live births) [10,11] are high in Nepal. EBF is estimated to prevent approximately 10% of child deaths and as such plays important role in meeting Nepal’s Millennium Development Goals (MDGs) 4 of reducing child mortality between 1996 and 2015 from 162 to 38 per 1,000 lives birth [4]. Therefore, it is important that EBF until six months of age is encouraged and supported by the health sector, families and communities in Nepal, however very little is known about the determinants of EBF in Nepal. A number of internationals studies have identified a number of socio-demographic determinants of EBF. Some of the most common factors found to be associated with EBF are: the economic status of family; education of mother; occupation of mother; utilisation of antenatanal care; place of residence; and access to information [12-15]. Similarly, the father’s socio-economic characteristics such as father’s education and occupation have also been reported to influence the EBF rates [16,17]. Updated knowledge about the determinants of EBF in Nepal could aid in the better design of infant nutrition programs. The objectives of the current study were to describe the changes in the proportion of EBF according to age for ≤5 months infants and to compare socio-economic and health factors associated with EBF in the 2006 and 2011 surveys.

## Methods

This study used data from the Nepal Demographic and Health Surveys (NDHS) collected in 2006 and 2011 [11,18]. The NDHS is nationally representative cross sectional study conducted every five years. It used a multistage cluster sampling design and internationally validated instruments.

For administrative purposes, Nepal is divided into 75 districts that are further divided into village development committees (VDC) and municipalities within the districts [11]. The VDC and municipalities are further divided into wards and sub-wards. For data collection purposes, the wards, sub-wards and groups of sub-wards were the primary sampling unit (PSU) in the rural areas. For urban areas, the wards were divided into sub wards to construct a PSU. A list of households was obtained for each PSU. The sampling procedure in 2006 and 2011 included two stages. In 2006 a total of 260 PSU were selected in stage one using a systematic sampling probability proportionate to the population size, with 82 urban and 178 rural PSU’s selected. In second stage, 30 urban households and 36 rural households were selected randomly for interview within each PSU. In the 2011 survey, the PSU were referred to as enumeration areas (EA). In first stage of sampling, a total of 289 EA were selected using a systematic sampling, including 95 urban and 194 rural EAs. In second stage, 35 households in urban and 40 household from rural areas were randomly selected from each EA for interview. Details of clustering and sample selection are explained in the respective reports [11,18].

### Sample size

In the 2006 survey [18], 9036 women aged 15–49 were included in the study. A total of 4397 men aged 15–59 were interviewed from every second selected household. For the 2011 survey, 12,674 women and 4,121 men aged 15–49 were interviewed [11]. The response rate was 96% in 2006 and 95.3% in 2011. Although a separate power calculation for under six months infants is not available in both surveys, we calculated the parameters based on the results of the current study to demonstrate that findings obtained from the study gained a reasonable power. A total of 482 mothers with infants under-six months of age from the 2006 survey and 227 from the 2011 survey were included in the analysis. Assuming a design effect of 2 in both surveys, based on Gpower [19], with an effective sample size of 241, the study power for the 2006 data was estimated as 90% to detect a difference of 32% in EBF rate between infants younger than 4 months and infants aged 5–6 months at 5% significance level. The study for the 2011 data with an effective sample size of 113 can detect a difference of 38% in EBF rate between infants younger than 4 months and infants aged 5–6 months with a power of 80% at 5% significance level.

### Study instruments

Three sets of questionnaires were used in the NDHS to collect information about:(i) the household; (ii) the women; and (ii) the men [11,18]. The household questionnaire asked basic information about all family members; household economic status and other environmental characteristics. The women’s questionnaire included background information (education, religion etc.), reproductive history, antenatal care, delivery and postnatal care, infant feeding, breastfeeding, immunization, child health, marriage, fertility, knowledge on HIV and other sexually transmitted diseases and access to health information [20]. The men’s questionnaire was shorter and included socioeconomic background (education, occupation), marriage, fertility, contraceptive practice, knowledge on HIV and other sexually transmitted diseases and access to health information [11]. Although the basic information contained in these questionnaires are similar across all countries using the DHS for consistency of comparison, a few modification are made for country specific information in respect to context and cultural backgrounds such as female genital mutilitation, HIV, tuberculosis, etc. These standard questionnaires were adapted to reflect national health and family planning programs unique to Nepal. The standard questionnaires in English were then developed in three different languages: Nepali, Bhojpuri and Maithili. The questionnaires were then backtranslated to English and neccesary changes were made if back translation did not match the original version [11,18].

### Data analysis

The rate of EBF was reported at less than one month, one, two, three, four, and five months to identify changes in the rate of EBF according to the age of the infant during the surveys. Categorical variables of interest associated with EBF were identified using Chi-square test (χ^2^). Association between the significant factors reported in bivariate analysis were then examined using multiple logistic regression controlling for potential confounders. Stepwise backward elimination procedure was used in the multiple logistic regression [21,22]. Crude and adjusted odds ratios (aOR) and their 95% CIs were reported. To monitor the change in the significant determinants of EBF between the 2006 and 2011 surveys, an additional multiple logistic regression model was built to assess if a particular factor significant in 2006 would still have a significant impact on EBF in 2011. The factors that were associated with the outcome variable at ≤0.25 in bivariate analysis were included in the multiple logistic regression model [22]. For the final model, a p-value < 0.05 was considered statistically significant. This analysis accounted for study design, and sample weight using Complex Sample Analysis [23]. The statistical analyses were conducted using Statistical Package for Social Science, Advanced statistics, Release 19.0 with add-on package (SPSS for windows, SPSS Inc., Chicago, IL, USA).

### Definition of variables

#### ***Outcome variable***

This study used EBF among infants aged ≤5 months as the outcome variable. The mothers who were currently breastfeeding and not providing other liquids or solids except for Oral Rehydration Solution (ORS), vitamins, mineral, medicine syrup were considered to be EBF. This outcome variable (EBF) was defined based on the WHO key infant feeding indicators and the guide to DHS statistics [20,24]. For our analysis, we categorized EBF as a dichotomous variable EBF =1; and Non-EBF = 0. The missing data on breastfeeding was treated as Non-EBF.

#### ***Independent variables***

Eastern, Central, Western, Mid-western and Far-western are the five development regions in Nepal. Based on the altitude, Nepal can also be divided into three ecological regions; Mountain, Hill and Terai (Plain) areas. Ethnicity was classified into three categories: relatively advantaged; relatively disadvantaged (Janjati); relatively disadvantaged (Dalit) [25,26]. The relatively disadvantaged (Janjati) group includes the indigenous groups who are socially and economically marginalized [26]. The relatively advantaged category included Brahmin, Chhetri, Thakuri and Sanyasi castes. Ethnicity was based on the caste system. There are 120 castes and languages in Nepal. It is complex to analyse the caste and ethnicity and their independent effect on health. The caste system is based on the Hindu scripture, *Manusmriti*[25]. The *Manusmriti* divides the population into different castes based on the occupation. This system has remained as primary identification of characteristics of Nepalese society since 1854 AD [27,28]. The Dalit community traditionally worked in leather works, carpentry, gold work, metal work, play musical instruments and perform other manual jobs. This group was socially excluded and systematically marginalized. Unfortunately, this kind of discrimination was supported by state law; the Muluki Ain (National Code) 1854 of Nepal. Later in 1963, the National Civil Code abolished this kind of discrimination by the state [27,28]. However, this culturally rooted system remains a major feature of current Nepalese society. The religions recorded in both the 2006 and 2011 surveys included: Hindu; Buddhist; Muslim; Kirat and Christian. Due to the small number of non-Hindu respondents, this variable was re-categorized into Hindu and Others. The Wealth Index is an international composite indicator of more than 40 asset variables [11,18]. It is categorised into poorest, poor, middle, rich and richest. Details of these indicators is presented elsewhere [11,20]. Father’s occupation was categorised into three categories: Agriculture: working in agriculture sector; Professional, clerical, service, and manual: office, business, clerical, skilled or unskilled jobs; Others (Not specified): occupations that were not specified to any of above groups. Timing of pregnancy was related to whether the last child was wanted or not wanted. It was categorised into: wanted, wanted later or not wanted. Prelacteal feeds was defined as any food provided to a newborn before introduction of mother’s milk [29]. It included sugar/salt solution, ghee, honey, etc. Infant’s size at birth was categorized based on mother’s perception into very small, small, average, large and very large. This was later re-categorized into three groups: small; average; or large [29] . Infant’s age was recoded into ≤4 months and 5 months as there is a cultural practice to introduce complementary feeding once the infant is about four/five months of age in Nepal.

### Ethics

The NDHS surveys were approved by Nepal Health Research Council, Nepal and ICF Macro Institutional Review Board in Calverton, Maryland, USA. Permission from Macro International (research agency) was obtained for the use of data which was de-identified prior to making it available for public use. Ethical approval from Curtin University Human Research Ethics Committee [protocol approval –SPH-16-2012] was also obtained for the data analysis.

## Results

### Description of socio-demographic factors

Table 1 describes the socio-demographic characteristics of the respondents in the 2006 and 2011 surveys. There were 482 mother-infant pairs (under six months) in the 2006 NDHS survey. The majority of mothers (67.2%) were between 20–29 years and 16.6% were teenage mothers. All were married. The majority of the mothers (87.1%) were Hindu. Almost half were from the Terai area (49.2%) and only 12.4% were from Mountainous areas. More than a half of the mothers (54.4%) had no formal education. Slightly less than half (46.4%) were from poor families.

**Table 1:** Proportion (%) of exclusive breastfeeding in infants 5 months of age and under by demographic and socioeconomic characteristics, Nepal 2006 (N = 482) and 2011 (N = 227)

**Factor**	**2006**			**2011**		
	**Total N [%]**	**EBF n [%]**^**#€**^	**p value**	**Total N [%]**	**EBF n [%]**^**#€**^	**p value**
**Infant’s age (months)**			<0.001			<0.001
<=4	380 (78.8)	218 (59.3)		187 (82.3)	134 (73.5)	
5-6	102 (21.2)	26 (33.1)		40 (17.7)	12 (33.1)	
**Age of Mother (years)**			0.730			0.361
<19	77 (16.0)	37 (47.6)		48 (21.0)	28 (68.9)	
20-29	324 (67.2)	168 (54.4)		139 (61.0)	98 (69.7)	
30-34	53 (11.0)	24 (54.2)		25 (10.9)	10 (54.1)	
35 and above	28 (5.8)	16 (56.9)		16 (7.0)	10 (47.6)	
**Development region**			0.150			0.448
Eastern	115 (23.9)	45 (45.1)		48 (21.2)	28 (60.4)	
Central	125 (25.9)	56 (47.9)		66 (28.9)	27 (72.0)	
Western	89 (18.5)	52 (62.9)		50 (22.0)	24 (55.3)	
Mid -Western	69 (14.3)	44 (65.0)		33 (14.6)	33 (70.7)	
Far-Western	84 (17.4)	48 (58.5)		30 (13.2)	34 (77.0)	
**Ecological region**			0.079			0.379
Mountain	60 (12.4)	22 (41.2)		15 (6.8)	24 (65.2)	
Hill	185 (38.4)	110 (60.9)		77 (34.1)	55 (59.7)	
Terai	237 (49.2)	113 (49.0)		134 (59.1)	67 (70.2)	
**Mother’s education**			0.009			0.531
No education	262 (54.4)	139 (54.2)		86 (38.1)	60 (71.9)	
Primary	83 (17.2)	47 (66.2)		55 (24.4)	36 (68.7)	
Secondary	113 (23.4)	51 (44.5)		68 (29.8)	38 (58.2)	
Higher	24 (5.0)	8 (27.9)		18 (7.7)	12 (62.7)	
**Mother’s occupation**			0.281			0.805
Not working	121 (25.1)	54 (47.9)		86 (38.1)	50 (69.5)	
Agriculture	290 (60.2)	159 (56.4)		122 (53.5)	84 (64.2)	
Working (paid)	71 (14.7)	32 (51.4)		19 (8.4)	12 (65.0)	
**Father’s occupation**			0.003			0.746
Agriculture	176 (36.5)	106 (63.4)		43 (18.9)	31 (67.6)	
Professional, clerical, service, and manual	195 (61.2)	134 (48.7)		168 (73.9)	105 (65.0)	
Others (Not specified)	11 (2.3)	5 (32.8)		17 (7.3)	10 (76.4)	
**Ethnicity**			0.036			0.296
Relatively advantaged	198 (41.9)	97 (48.2)		93 (41.2)	65 (66.2)	
Relatively disadvantaged (Janjati)	155 (32.8)	86 (62.4)		74 (32.6)	42 (59.2)	
Relatively disadvantaged (Dalit)	119 (25.2)	56 (47.0)		59 (26.2)	39 (75.4)	
**Religion**			0.128	n=		0.221
Hindu	420 (87.1)	211(51.2)		190 (83.8)	125 (68.2)	
Others	62 (12.9)	34 (65.5)		37 (16.2)	21 (56.4)	
**Sex of child**			0.078			0.086
Male	250 (51.9)	137 (57.2)		118 (52.0)	73 (60.3)	
Female	232 (48.1)	108 (48.6)		109 (48.0)	73 (72.4)	
**Wealth index**			0.015			0.310
Poorest	138 (28.6)	88 (67.2)		52 (22.8)	39 (61.7)	
Poor	86 (17.8)	42 (51.2)		45 (20.0)	31 (65.5)	
Middle	94 (19.5)	44 (54.3)		58 (25.5)	31 (77.4)	
Richer	92 (19.1)	43 (46.8)		44 (19.2)	30 (69.1)	
Richest	72 (14.1)	28 (40.6)		28 (12.5)	15 (49.1)	

^#^the proportion represents the percentage after adjusting for study design and sample weight. This may not be equal to simple calculation of proportion. ^€^: the percentage in the cells is the row percentage of the each sub groups.

There were a total of 227 mother-infant pairs (under six months) in the 2011 NDHS survey. The majority of mothers (61.0%) were between 20–29 years and 21.0% were teenage mothers. Almost all (99.6%) were married. The majority of the mothers (83.8%) were Hindu. More than half (59.1%) were from the Terai area and only 6.8% were from the Mountainous areas. Slightly more than one third (38.1%) of the mothers had no formal education. Slightly less than half (47.0%) were from poor or very poor families (Table 1).

### Description of health related factors

Table 2 describes the health related characteristics of the respondents for 2006 and 2011. In the 2006 survey, 30.4% were first time mothers and 13.7% of mothers had a birth interval of less than two years. Only 31.7% had had four or more antenatal care visits during their pregnancy. Less than two-thirds of mothers had taken iron supplements at some point of time during their pregnancy. Only 21.6% infants were delivered at health facilities. Slightly more than a third (36.2%) of the mothers had provided prelacteal feeds to their newborn. One in five (18.3%) of infants were smaller than average size as perceived by the mothers.

**Table 2:** **Proportion (%) of exclusive breastfeeding in infants** 5 **months of age and under by health related characteristics, Nepal 2006 (N = 482) and 2011 (N = 227)**

**Factor**	**2006**			**2011**		
	**Total N [%]**	**EBF n [%]**^**#€**^	**p value**	**Total N [%]**	**EBF n [%]**^**#€**^	**p value**
**Parity**			**0.002**			**0.422**
First	146 (30.4)	58 (41.3)		80 (35.1)	51 (66.7)	
Second or third	224 (46.6)	123 (57.2)		102 (44.9)	65 (70.1)	
Fourth or more	111 (23.1)	64 (63.2)		45 (20.0)	30 (57.1)	
**Birth interval by month**			0.001			0.989
No previous birth	147 (30.5)	58 (40.9)		80 (35.1)	51 (66.7)	
< 24 months	66 (13.7)	37 (63.1)		41 (18.0)	20 (67.1)	
> = 24 months	269 (55.8)	150 (58.2)		106 (46.9)	75 (65.7)	
**Types of pregnancy**			0.293			N/A
Single	472 (97.9)	241 (53.7)		225 (99.1)		
Twin/Multiple	10 (2.1)	4 (33.0)		2 (0.9)		
**Timing of pregnancy**			0.019			0.314
Wanted	312 (64.7)	162 (53.0)		154 (67.9)	103 (69.4)	
Wanted later	89 (18.5)	36 (42.1)		41 (18.0)	25 (68.0)	
Not wanted	81 (16.8)	47 (66.1)		32 (14.0)	18 (49.2)	
**ANC (number of visits)**			0.108			0.093
No visit	100 (21.0)	58 (63.4)		28 (12.2)	17 (84.7)	
1-3	226 (47.4)	111 (47.0)		87 (38.3)	52 (66.8)	
4 or more	151 (31.7)	74 (57.6)		112 (49.4)	77 (61.4)	
**Deworming**			0.223			0.447
No	332 (68.9)	178 (55.7)		136 (59.9)	90 (63.7)	
Yes	150 (31.1)	67 (48.3)		91 (40.1)	56 (70.2)	
**Iron tablet or syrup consumption**			0.024			0.995
No/Do not know	161 (33.4)	95 (62.0)		41 (18.0)	22 (66.3)	
Yes	321 (66.6)	150 (49.6)		186 (82.0)	124 (66.3)	
**Mode of delivery**			0.010			0.030
Vaginal	468 (97.1)	242 (54.6)		209 (92.2)	134 (64.5)	
Caesarean	14 (2.9)	3 (18.1)		18 (7.8)	12 (87.6)	
**Place of delivery**			0.004			0.362
Home	378 (78.4)	201(57.2)		124 (54.4)	70(63.3)	
Health facility	104 (21.6)	44 (39.7)		103 (45.6)	76 (69.9)	
**Size of the infant** (Perceived by mothers)			0.016			0.283
Average Size	282 (58.6)	144 (50.9)		148 (65.7)	91 (61.6)	
Small	88 (18.3)	51 (46.3)		41 (18.4)	28 (73.6)	
Large	111 (23.1)	50 (67.5)		36 (15.9)	26 (75.0)	
**Post-natal care of the infant**			0.383			0.866
No	453 (94.0)	231 (52.5)		109 (48.1)	67 (65.5)	
Yes	29 (6.0)	14 (62.5)		118 (51.9)	79 (67.0)	
**Prelacteal feeds**			0.003			0.747
Given something	172 (36.2)	73 (43.1)		65 (28.5)	33 (64.3)	
Given nothing	303 (63.8)	170 (60.5)		162 (71.5)	113 (67.1)	

^#^the proportion represents the percentage after adjusting for study design and sample weight. This may not be equal to simple calculation of proportion. ^€^: the percentage in the cells is the row percentage of the each sub groups. The N/A: not application for chi square test.

In the 2011 survey, 35.1% of the mothers were the first time mothers and 18.0% of mothers had a birth interval less than two years. More than a half (56.3%) had had four or more antenatal care visits during their pregnancy. The majority (82.0%) of mothers confirmed that they had taken iron supplementation during pregnancy. Less than half of the deliveries (45.6%) were conducted in health facilities. Almost one in three (28.5%) mothers had introduced prelacteal feeds. One in every five (18.4%) of infants were smaller than average size as perceived by the mothers (Table 2).

### Comparison of Exclusive breastfeeding during 2006 and 2011

Half of the mothers (53.2%; 95% CI (47.1%-59.3%); design effect: 1.842) with infants aged 0–5 months had EBF their infant within the last 24-hours of survey in 2006. More mothers (66.3%; 95% CI (56.6%-74.8); design effect: 2.141) had EBF their infants within the past 24 hours in the 2011 survey.

### Determinants of EBF, NDHS 2006 and 2011

Table 3 shows the factors associated with providing EBF in 2006. In 2006, after controlling for development region, mother’s education, ethnicity, religion, sex of child, wealth quintile, birth interval, timing of pregnancy, number of ANC visits, deworming, iron consumption during pregnancy, mode of delivery, size of infants and prelacteal feeding; infant’s age, ecological regions, father’s occupation, parity, and place of delivery were significantly associated with providing EBF. Infants of ≤4 months were more likely [aOR 3.083; 95% CI (1.825-5.206)] to have been EBF than older infants of 5 months. Infants from the Hills [aOR 3.426; 95% CI (1.568-7.474)] were more likely to have been EBF than those from the Mountain region. Infants whose fathers were from professional, clerical, service, and manual occupations were less likely [aOR 0.578; 95% CI (0.383-0.873)] to have been EBF. High parity mothers were more likely to EBF than first time mothers with an increase in the odds of providing EBF by second or third time mothers [aOR 1.786; 95% CI (1.076-2.996)] and fourth-time mothers or more [aOR 1.968; 65% CI (1.020-3.799)]. The mothers who delivered in home were more likely to EBF than their counterparts delivering in health facilities [aOR 1.886; 95% CI (1.044-3.407)].

**Table 3:** **Determinants of exclusively breastfeeding in infants** 5 **months of age and under - unadjusted and adjusted Odds Ratio, Nepal 2006**

**Factor**	**Total N [%]**	**EBF n [%]**	**Crude OR**	**95% CI**	**Adjusted OR**	**95% CI**
**Infant’s age (months)**			P < 0.001		P < 0.001	
<=4	380 (78.8)	218 (59.3)	2.946	1.898- 4.573	3.083	1.825 (5.206)
5-6	102 (21.2)	26 (33.1)	1.00		1.00	
**Ecological region**			P = 0.109		P = 0.009	
Mountain	60 (12.4)	22 (41.2)	1.00		1.00	
Hill	185 (38.4)	110 (60.9)	2.221	0.992-4.946	3.426	1.568-7.474
Terai	237 (49.2)	113 (49.0)	1.370	0.670-2.800	1.841	0.942-3.559
**Father’s occupation**			P = 0.082		P = 0.018	
Agriculture	176 (36.5)	106 (63.4)	1.00		1.00	
Professional, clerical, service, and manual	195 (61.2)	134 (48.7)	0.546	0.388-0.769	0.578	0.383-0.873
Others (Not specified)	11 (2.3)	5 (32.8)	0.281	0.061-1.177	0.286	0.089-0.919
**Parity**			P = 0.001		P = 0.045	
First	146 (30.4)	58 (41.3)	1.00		1.00	
Second or third	224 (46.6)	123 (57.2)	1.905	1.242-2.921	1.786	1.076-2.996
Fourth or more	111 (23.1)	64 (63.2)	2.442	1.427-4.180	1.968	1.020-3.799
**Place of delivery**			P = 0.004		P = 0.036	
Home	378 (78.4)	201(57.2)	2.025	1.261-3.253	1.886	1.044-3.407
Health facility	104 (21.6)	44 (39.7)	1.00		1.00	

Independent variables included in the initial model: age of infant, development region, ecological region, mother’s education, father’s occupation, ethnicity, religion, sex of child, wealth quintile, parity, birth interval, timing of pregnancy, number of ANC visits, deworming, iron consumption during pregnancy, mode of delivery, place of delivery, size of infant, and prelacteal feeding. Only significant variables in backward elimination procedure are presented in the table. Valid case: 481.

Similar analysis was conducted for the 2011 NDHS survey data (Table 4). After controlling for infant’s sex, religion, ANC visits and mode of delivery, the infant’s age remained significantly associated with providing EBF. Infants of ≤4 months were more likely [aOR 6.11; 95% CI (2.349-15.897)] to have been EBF when compared to older infants of 5 months.

**Table 4:** **Determinants of exclusively breastfeeding in infants** 5 **months of age and under, Nepal 2011**

**Factor**	**Total N [%]**	**EBF n [%]**	**Crude OR**	**95% CI**	**Adjusted OR**	**95% CI**
**Infant’s age (months)**			P < 0.001		P < 0.001	
<=4	187 (82.3)	134 (73.5)	5.690-	2.161-14.554	6.11	2.349 -15.897
5-6	40 (17.7)	12 (33.1)	1.00		1.00	
**Sex of child**			P = 0.088		P = 0.471	
Male	118 (52.0)	73 (60.3)	1.00		1.00	
Female	109 (48.0)	73 (72.4)	1.704	0.923-3.146	1.290	0.643-2.589
**Religion**			P = 0.224		P = 0.229	
Hindu	190 (83.8)	125 (68.2)	1.685	0.731-3.760	2.082	0.869-4.990
Others	37 (16.2)	21 (56.4)			1.00	
**ANC (number of visits)**			P = 0.053		P = 0.119	
No visit	28 (12.2)	17 (84.7)	1.00		1.00	
1-3	87 (38.3)	52 (66.8)	0.362	0.107-1.226	0.435	0.126-1.507
4 or more	112 (49.4)	77 (61.4)	0.286	0.081-1.017	0.346	0.091-1.317
**Mode of delivery**			P = 0.042		P = 0.150	
Vaginal	209 (92.2)	134 (64.5)	0.257	0.069-0.954	0.349	0.083-1.474
Caesarean	18 (7.8)	12 (87.6)	1.00		1.00	

Independent variables entered in the initial model: infant’s age, sex of infant, religion, ANC visits, and mode of delivery.

The 2006 and 2011 surveys were also compared to monitor any change in the determinants of EBF (Table 5). An additional multiple logistic regression model was built using the 2011 survey data taking the significant determinants of EBF from the 2006 survey data (infant’s age, ecological region, father’s occupation, parity and place of delivery) as independent variables. This new model also revealed that only the infant’s age remained statistically significant factor of EBF for 2011 survey data (Table 5, Model 1^b^).

**Table 5:** Comparison of the determinants of exclusive breastfeeding in infants 5 months of age and under between 2006 and 2011, Nepal

**Factors**	**Survey 2006**		**Survey 2011**			
	**Model 1**		**Model 1**^**b**^		**Model 2**	
	**Adjusted OR**	**95% CI**	**Adjusted OR**	**95% CI**	**Adjusted OR**	**95% CI**
**Infant’s age (months)**	P < 0.001		**p < 0.001**		P < 0.001	
<=4	3.083	1.825 (5.206)	9.449	3.375-26.459	6.11	2.349 -15.897
5-6	1.00		1.00		1.00	
**Ecological region**	P = 0.009		p = 0.648		N/S	
Mountain	1.00		1.00			
Hill	3.426	1.568-7.474	0.512	0.151-1.731		
Terai	1.841	0.942-3.559	1.324	0.393-4.459		
**Father’s occupation**	P = 0.018		P = 0.668		N/S	
Agriculture	1.00		1.00			
Professional, clerical, service, and manual	0.578	0.383-0.873	0.559	0.213-1.470		
Others (Not specified)	0.286	0.089-0.919	1.409	0.293-6.745		
**Parity**	P = 0.045		P = 0.325			
First	1.00		1.00			
Second or third	1.786	1.076-2.996	1.304	0.612-2.777		
Fourth or more	1.968	1.020-3.799	0.586	0.201-1.709		
**Place of delivery**	P = 0.036		P = 0.269		N/S	
Home	1.886	1.044-3.407	0.661	0.316-1.382		
Health facility	1.00		1.00			

Independent variable entered in model 1: age of infant, development region, ecological region, mother’s education, father’s occupation, ethnicity, religion, sex of child, wealth quintile, parity, birth interval, timing of pregnancy, number of ANC visits, deworming, iron consumption during pregnancy, mode of delivery, size of infant, and prelacteal feeding. Only significant variables in backward elimination procedure are presented in the table. Valid case: 481. Model 1^b^: This model included the significant variables of Model 1(2006) to allow comparability. Model 2: Independent variables entered in the model 2: infant’s age, sex of infant, religion, ANC visits, and mode of delivery. N/S: Not significant.

Infant’s age has been consistently reported as an associated factor for EBF [14,15,30], and confirmed by our study. The infant’s age was the only significant factor found in the 2011 data suggesting the possibility that the effect of other factors on EBF may be confounded due to the influence of age on EBF. For 2006 survey data, this additional model revealed that ecological region, father’s occupation, sex of child, wealth quintile, parity, number of ANC visits, iron consumption during pregnancy, mode of delivery and place of delivery were significantly associated with EBF when age of infant was excluded from the initial model of Table 3. The infants whose mothers were from Hills [aOR 4.860; 95% CI (2.096-11.271)], and Terai [aOR 3.198; 95% CI (1.438- 7.118)]; attended four or more ANC visits [aOR 3.810; 95% CI (1.385-10.482)], and had vaginal (normal) delivery [aOR 4.403; 95% CI (1.173-16.524)] were more likely to be EBF. On the other hand, the infants whose fathers were from professional, clerical, service, and manual [aOR 0.642; 95% CI (0.412-1.00)] and other occupation [aOR 0.221; 95% CI (0.049-0.987)] were less likely to provided EBF. The infants with wealthier mothers were less likely to be provided with EBF. While there was a significant change in factors associated with EBF in 2006, excluding infant’s age from 2011 data (Table 4) showed that there was no change in the factors associated with EBF in 2011 (Table not shown).

### Changes in the rate of exclusive breastfeeding according to infant’s age, Nepal 2006 and 2011

Table 6 describes the gradual decline in the rate of EBF according to infant’s age (in months) during the survey. The rate of EBF at less than one month of age was 90.6% in 2006 and 78.4% in 2011. Not surprisingly, the EBF rate decreased as the age of infant increased and it declined to 33.1% in 2006 and 33.1% in 2011 at five months. The 2011 EBF rate at age 1, 2, 3, 4, and 5 months was higher than that in 2006; however, there were no statistically significant differences in EBF rates between 2006 and 2011 with the exception at 4 months [32.0% CI (19.9-47.0) for 2006; 65.5% CI (48.1-79.6) for 2011]. It should be noted that even when the demographic surveys in Nepal included a large household level sample, it was not a large enough sample to make comparisons within age strata for EBF. The rate of EBF; therefore, has a wide 95% confidence interval showing variation due to the small number of observation in particular age strata. For comparison purpose, the rates of EBF for each age group were calculated using the number of infants breastfed exclusively.

**Table 6:** **Proportion (95% CI) of exclusive breastfed infants by age**^**©**^**, Nepal 2006 and 2011**

**Infant’s age**	**NDHS 2006**		**NDHS 2011**	
	**N**	**Prevalence (95% CI)**^**#**^	**N**	**Prevalence (95% CI)**^**#**^
< one	52	90.6 (79.2-96.1)	15/17	78.4 (20.0-98.1)
One	68	85.3 (76.0-91.3)	32/34	86.1 (73.3-93.3)
Two	74	53.4 (41.8-64.7)	43/36	74.8 (57.0-87.0)
Three	87	53.5 (40.7-65.9)	47/50	70.0 (57.0-81.2)
Four	99	32.0 (19.9-47.0)*	50/49	65.5 (48.1-79.6)*
Five	102	33.1 (23.8-43.9)	40/36	33.1 (19.1-50.8)
***<six months***	***482***	***53.2 (47.1-59.3)***	***227***	***66.3 (56.6-74.8)***

^#^the proportion represents the percentage after adjusting for study design and sample weight. This may not be equal to simple calculation of proportion. ^©^The age of the infant represents the age at the time of survey. *Statistically significant difference at p < 0.05.

## Discussion

Nutrition in early childhood is essential not only for the immediate health of the child but also for the long term benefits. EBF confers protection against infectious diseases such as gastroenteritis and respiratory illness as well as other chronic diseases in the long term [31] while preventing up to 10% of all child deaths. Therefore, WHO and UNICEF recommend exclusive breastfeeding for the first six months and continued breastfeeding thereafter [1,2]. To reach the Millennium Development Goal targets for child survival, Nepal needs to reduce infant mortality from 108 per 1000 live births in 1990 to 34 per 1000 live births by 2015 [32]. The infant mortality was 46 per 1000 live births in Nepal in 2011 [11]. This reduction from 46 to 34 (in 2015) can in part be achieved through a greater adoption of the recommended breastfeeding practice.

In this study, only 66.3% in 2011 and 53.2% in 2006 of infants were EBF within 0–5 months. While it is encouraging to note that there was a 13% increase in the rate of EBF it is still significantly lower than the WHO/UNICEF recommended level of 90% [2,12,14]. Nevertheless, the EBF rate among ≤5 months infants is higher in Nepal compared to Bangladesh (46%), India (47%) and Pakistan (50%) [33]. When examined for the age specific changes in the rate of EBF, the data showed a rapid decline in the EBF around the age of four months which is similar to the findings from India [17] and Pakistan [34]. At the age of five months, the rate of EBF in Nepal had dropped to 33.1% in 2011. Our findings suggest that achieving the WHO/UNICEF target of EBF for six months is challenging.

The infant’s age, ecological region, father’s occupation, parity, and place of delivery significant determinants in 2006 for providing EBF; however, only the infant’s age was significantly associated with EBF in 2011. When age of infant was removed from the model, wealth status, ANC visits, sex of infant, iron consumption during pregnancy, and mode of delivery were significant determinants of providing EBF in 2006.

The increase in the rate of EBF amongst younger infants (≤4 months) could in part be attributable to the changes in child health programs implemented in Nepal since 2006. Childhood and newborn illness management programs such as the Community Based Integrated Management of Childhood Illness (CB-IMCI) and Community Based Newborn Care Program (CB-NCP) are two major programs that focus on breastfeeding [35,36]. The CB-IMCI program was implemented nationwide in 2008 [32]. CB-NCP program started in 2007 and has been underway to scale up gradually to all 75 districts in Nepal [37]. These programs recommend that the health workers assist mothers by providing knowledge and skills to breastfeed. Such support from the health workers would encourage mothers to exclusively breastfeed their infants for longer.

The association of father’s occupation with EBF has been inconsistent [17,38]. This study found similar inconsistencies, whereby father’s occupation was associated with EBF in 2006 but not in 2011. Fathers who work in professional, clerical, service, and manual occupations usually go outside the home in search of work and the burden of work may be higher for the women in the household leaving less time for them to EBF the infants. There have been no studies that have examined the effect of father’s occupation on breastfeeding in Nepal. Father’s increased knowledge and being in a paid occupation have been found to have a positive impact on breastfeeding in other countries [39-41]; however, in this study the infants whose fathers were from agriculture occupation were likely to be EBF than the others. The father’s impact of EBF is an area of increasing research in developed countries [41] and is an area that needs further exploring in developing countries including Nepal, as a potential contributor to improving EBF rates.

Parity was a significant predictor of providing EBF in the 2006 survey. It was shown that the mothers who have had previous child birth were more likely to provide EBF compared to the first time mothers. The multiparous mothers have already had experience in breastfeeding and maybe better able to manage EBF with their other household tasks [14,42]. The ecological differences in EBF practices found in this study may be an important consideration in the context of Nepal. People in the Mountain regions live a life characterised by greater hardship due to extreme cold weather, harder physical work, and less access to basic services [11]. This might leave mothers less time to exclusively breastfeed their child. The geographic differences have been shown to have an impact on EBF practices in Sri Lanka [43] and other countries [12,34]. This study also found that female infants were less likely to be EBF. There is a strong gender preference for sons within South Asian cultures and it has been suggested that that boys might be given more time for infant feeding than the girls [44,45]. These cultural factors might impact EBF adversely.

Obtaining antenatal care (ANC) services for mother is the first step in the continuum of care for mother and child. ANC visits provide mothers an opportunity to discuss with health workers their problems and other health issues. It also gives an excellent opportunity for health workers to educate mothers about nutrition and EBF. The time lag between antenatal visits and initiation of EBF, of course, is long; however, different studies have found an association of ANC and exclusive breastfeeding practices. Our study reported that the mothers who had four or more ANC visits were more likely to have EBF their infant. This finding is similar to other studies from Egypt [46], Ghana [47], and Nigeria [12].

In this study, vaginal (normal) delivery was associated with higher EBF practice. Similar finding was reported from a Vietnamese study where the mothers who delivered normally (without requiring caesarean section) were more likely to exclusively breastfeed children [48]. Mode of delivery might have effect on breastfeeding in several ways. The mothers who have been undergoing surgical procedure might not feel well to breastfed after surgery, or might be under influence of anaesthesia. Mother’s difficulty in holding baby due to fresh wound might be another barrier to breastfeed successfully [42].

Mothers who delivered in home were more likely to EBF than their counter parts delivering in health facility. The vast majority (>80%) of childbirth in 2006 survey occurred at home. The mothers who are highly educated, who lived in the city and who are from higher wealth section of the populations may deliver at the health facility [18]. In this study, wealthier mothers were less likely to EBF their infants. There have been several studies which report and argue that the mothers who are poor are more likely to EBF as they do not have other option such as infant formula [12,13]. Due to intensive marketing of milk substitutes, mothers who are comparatively well-off may feel encouraged to introduce such breast milk substitute [13]. Nevertheless, within the Nepalese society, breastfeeding has always been a universal practice and traditionally it has always been considered one of the most important factors that established the relationship between mother, child and siblings [49]. Therefore, the mothers who delivered in home may strictly follow the cultural practice of breastfeeding.

There are several strengths of this study. It is based on data from national demographic and surveys that used internationally validated questionnaires, it applied international EBF definitions combined with a strong methodology [50]. It had a high response rates (>94%) indicating generalizability of the research findings to the entire nation. The current study provides a comparison of the 2006 and 2011 surveys and examines the changes in the determinants of EBF in Nepal which has not been reported before. This comparison gives indication for future interventions and provides a benchmark for future comparisons. This study also suggests methodological consideration by using different ways of examining the determinants of EBF. Like most observational studies, this study has some limitations. The cross sectional nature of the study prevents it from making causal inferences. All the information in NDHS surveys are based on interviews; it is possible that some responses could suffer from recall bias and/or socially desirable responses [51]; however, interview method is most widely used method for collecting information on breastfeeding. Another major limitation of NDHS is 24-hour recall of information. It has been reported previously that such 24-hour period prevalence of EBF is likely to overestimate the EBF rate [52]. Cohort studies with repeated measure are recommended to report a more accurate rate of exclusive breastfeeding for six months [52]. The small sample size is a major issue in current NDHS studies to report EBF rate. It is possible that due to smaller number of sample size in 2011 a number of important independent variables were not significant. A reasonable improvement in NDHS can be achieved by including large enough sample for each month of age of the infant so that findings could be relied on to report the rate of EBF by infant’s age. Further improvement could be made by establishing sentinel sites to report EBF rate by age and complement the NDHS data [52].

## Conclusions

Half of the infants 0–5 months (53.2%) in 2006 and 66.3% in 2011 were exclusively breastfed. There was increase in the rate of EBF from 2006 to 2011. However, the 2011 EBF rate is still far below the WHO recommended goal of 90% [2]. The current study suggests the need for further interventions to promote exclusive breastfeeding. Antenatal counselling could be feasible approach that can improve the EBF practices in Nepal [53-57]. The infant’s age was a consistent determinant of EBF in both 2006 and 2011. Mothers could be supported/encouraged to maintain breastfeeding till 6 months and provided with skills through existing primary health care outreach clinics (held 2–5 clinics each month) and immunization clinics (held 3–5 clinics each month) in the local communities [58]. Given the current >90% routine immunization coverage in Nepal, reaching the majority of the mothers for breastfeeding promotion programs through immunization clinics to maintain breastfeeding until 6 months is a feasible option that needs further consideration [58]. These outreach clinics (immunization clinics and primary health care) are run by village health workers and maternal and child health workers. To promote EBF to 6 months through these groups of health workers, health program managers should consider capacity building to provide them with the necessary skills to support mothers.

Current child health promotion programs in Nepal focus on the mothers. Involving fathers and other significant relatives in the family unit, including the mothers-in-law, may also improve the rate of EBF. A study from Nepal has suggested that the younger females (daughters-in-law) were expected to follow the decision made by their mothers-in-law about attendance to antenatal care and other maternal and child health issues [59]. Older women in Nepal such as mothers-in-law generally have less education than their daughters-in-law [59] and therefore, breastfeeding promotion programs should include them in the programs as a method of increasing the overall EBF rate. Educating fathers has been found beneficial in increasing breastfeeding practice elsewhere [41,60] and it is an important new area of intervention to be considered in Nepal. Fathers who are involved in decision making can support their wife’s decision to breastfeed for longer. These results have highlighted some key social determinants which could be relevant to increasing EBF outcomes at six months and beyond; and made recommendation for further action which would warrant further investigation.

## Competing interests

The authors declare that they have no competing interests.

## Authors’ contributions

VK, KS and YZ contributed in the concept of analysis of NDHS dataset. VK performed statistical analysis. VK wrote manuscript with significant contribution from KS. YZ supervised the analysis, and interpretation of the results, and edited the manuscript. All of the authors contributed in revision and agreed on the final manuscript.

## Authors’ information

VK holds an MPH degree. He has been working in child health programs in Nepal for more than five years. Newborn care and child nutrition is the focus of his work in Nepal and MPH studies. Yun Zhao is a senior lecturer in the School of Public Health and teaches in the postgraduate programs. She has an MSc and PhD in statistics. Kay Sauer is a senior lecturer in the School of Public Health and coordinates the MPH/DrPH programs. She has an MSc and PhD in Behavioural Sciences.

## Pre-publication history

The pre-publication history for this paper can be accessed here:

http://www.biomedcentral.com/1471-2458/13/958/prepub
